# Matrix metalloproteinase-8 rs11225395 polymorphism correlates with colorectal cancer risk and survival in a Chinese Han population: a case-control study

**DOI:** 10.18632/aging.103930

**Published:** 2020-10-14

**Authors:** Jiandong Tai, Di Sun, Xu Wang, Zhenhua Kang

**Affiliations:** 1 Department of Colorectal and Anal Surgery, The First Hospital of Jilin University, Changchun 130021, Jilin, China

**Keywords:** colorectal cancer, MMP-8, polymorphism, case-control study, survival, susceptibility

## Abstract

Matrix metalloproteinase-8 (MMP-8) is a gene associated with inflammation and prognosis in colorectal cancer (CRC). Here, we studied the link between the rs11225395 polymorphism of MMP-8 gene and CRC risk. We recruited 551 CRC cases and 623 controls from among a subpopulation of Han Chinese patients. Data found that this variant was connected to an increased risk of CRC (TT versus CC: OR, 1.76; 95%CI, 1.09–2.84; *P* = 0.021; T versus C: OR, 1.29; 95%CI, 1.07–1.56; *P* = 0.007). Stratified analyses indicated a positive association among smokers (TT versus CC: OR, 2.31; 95%CI, 1.12–4.79; *P* = 0.024), males, and patients ≥ 60 years old. Crossover analysis showed that the potential interaction between smoking or drinking and the MMP-8 rs11225395 polymorphism was related to elevated risk for CRC. The rs11225395 polymorphism was also connected with lymph node metastasis and TNM stage. Moreover, the CRC cases carrying a TT genotype of MMP-8 rs11225395 presented had poorer overall survival than the CC genotype carriers. These findings show that MMP-8 rs11225395 correlates with an elevated risk of CRC and poor patient prognosis in a subpopulation of the Han Chinese subpopulation. Thus, the MMP-8 rs11225395 polymorphism could potentially function as a biomarker predictive of CRC susceptibility.

## INTRODUCTION

Colorectal cancer (CRC) is one of the most deadly cancers, causing about 900,000 cancer related deaths every year [1, 2]. The number of CRC patients is projected to increase to more than 2.2 million by 2030 [3]. CRC ranks as the fifth and fourth most prevalent cancer among men and women in China, respectively [4]. The incidence and mortality among women are ~25% lower than those in men [1]. Nationwide screening programs, improved lifestyle and diet, and increased colonoscopy screenings have contributed to the decreasing trend in the incidence of this disease [5]. CRC patients show classical signs and symptoms including altered bowel habits, occult or overt rectal bleeding, anemia, or abdominal pain [6]. Unfortunately, CRC pathogenesis is poorly understood and only becomes symptomatic at advanced stages, with surgery currently being the most effective treatment. Genetic predisposition, unhealthy lifestyles, obesity, and other environmental factors such as smoking and drinking corelate with the development of CRC [1, 6, 7]. Genome wide association studies (GWASs) revealed a host of novel risk loci for CRC patients [8–11].

Matrix metalloproteinases (MMPs), zinc and calcium-dependent proteolytic enzymes could degrade extracellular matrix proteins and components. Some MMPs are related with cancer progression, metastasis and invasion [12, 13]. MMP-8 is an enzyme in the connective tissue that is primarily produced by neutrophils and that cleaves collagens, growth factors, cell adhesion proteins, and cytokines [14]. Increased MMP-8 levels in CRC correlated with disease progression and inflammation [15]. Böckelman et al. showed that MMP-8 levels served as prognostic biomarker for CRC [16]. In addition, Sirniö et al. showed that high MMP-8 serum levels corelated with decreased survival in CRC [17]. Elevated MMP-8 levels were also observed in CRC patients who developed anastomotic leakage after surgery [18].

The MMP-8 gene is shown to locate on chromosome 11q22.2. Rs11225395 polymorphism is in the promoter region of MMP-8 gene. The T allele of the rs11225395 polymorphism resulted in increased protein expression of MMP-8 [19]. Several studies addressed the link between the rs11225395 polymorphism of MMP-8 gene and different cancer risk, but with conflicting findings [20–31]. Here, we aimed to explore the relationships between both CRC risk and survival prognosis and the MMP-8 rs11225395 polymorphism.

## RESULTS

### Population characteristics

Table 1 summarizes the clinicopathological characteristics and data of all included patients. The frequencies of gender, age, smoking and drinking showed no obvious differences between these two groups. In this study, 551 CRC patients and 623 controls were recruited. In terms of tumor site, CRC patients were divided into two groups: the rectum (66.9%) and colon (33.1%). Adenocarcinoma accounted for the most common pathology subtype (95.1%). Other relevant data are presented in Table 1.

**Table 1:** Patient demographics and risk factors in colorectal cancer.

**Characteristics**	**Case (N=551)**	**Control (N=623)**	***P***
Age	61.86±12.62	60.40±13.33	0.055
Sex			0.249
Male	341(61.9%)	365(58.6%)	
Female	210(38.1%)	258(41.4%)	
Smoking			0.149
Yes	315(57.2%)	330(53.0%)	
No	236(42.8%)	293(47.0%)	
Alcohol			0.333
Yes	303(54.9%)	325(52.2%)	
N0	248(45.1%)	298(47.8%)	
BMI	22.24±2.79	22.54±2.88	0.066
CRP, mg/dL			
<0.4	315(57.2%)	593(95.2%)	
≥0.4	236(42.8%)	30(4.8%)	
ESR, mm/hr			
<15	303(55.0%)	597(95.8%)	
≥15	248(45.0%)	26(4.2%)	
Family History			
Yes	117(21.2%)		
No	434(78.8%)		
Histological grade			
well differentiated	77(13.9%)		
Moderate differentiated	406(73.7%)		
Poor differentiated	68(12.4%)		
TNM stage			
I+II	234(42.5%)		
III+IV	317(57.5%)		
Tumor size			
>5 cm	321(58.3%)		
≤5 cm	230(41.7%)		
Lymph node metastasis			
No	318(57.7%)		
Yes	233(42.3%)		
Histology			
Adenocarcinoma	524(95.1%)		
Squamous cell carcinoma	22(4.0%)		
Others	5(0.9%)		
Location of colorectal cancer			
colon cancer	182(33.1%)		
rectal cancer	369(66.9%)		

TNM, tumor node metastasis; BMI, body mass index; CRP, C-reactive protein; ESR, erythrocyte sedimentation rate.

### Association of MMP-8 gene rs11225395 polymorphism with the risk of CRC

**Figure 1:**
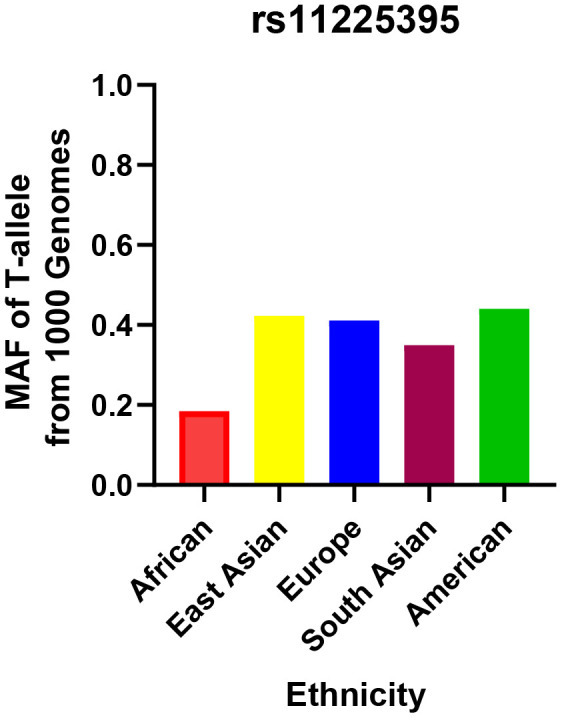
**Minor allele frequencies for MMP-8 rs11225395 polymorphism in controls, stratified by ethnicity.**

The relationship between this locus and CRC risk is presented in Table 2. The genotype distributions in control groups were in accordance with HWE test (*P* > 0.05). The minor allele frequency (MAF) of the rs11225395 polymorphism for CRC cases was 0.27. In addition, we summarized the MAF for different populations around the world in Figure 1, which was obtained from the 1000 Genomes repository [33]. The TT or CT+TT genotypes were shown to increase the susceptibility to CRC (TT vs. CC: OR, 1.76; 95%CI, 1.09–2.84; *P* = 0.021). The associations remained statistically significant even after adjusting for gender and age. Additionally, the T allele was shown to increase the risk of CRC.

**Table 2:** Logistic regression analysis of associations between MMP-8 rs11225395 polymorphism and risk of colorectal cancer.

**Genotype**	**Cases^a^(n=551)**		**Controls^a^(n=623)**		**OR (95% CI)**	***P***	*OR (95% CI)	*P
	**n**	**%**	**n**	**%**				
rs11225395C/T								
CC	287	52.2%	367	59.0%	1.00	-	-	-
CT	219	39.8%	223	35.9%	1.26(0.99-1.60)	0.065	1.25(0.98-1.59)	0.073
TT	44	8%	32	5.1%	**1.76(1.09-2.84)**	**0.021**	**1.76(1.09-2.85)**	**0.022**
CT+TT	263	47.8%	255	41%	**1.32(1.05-1.66)**	**0.019**	**1.31(1.04-1.66)**	**0.021**
CC+CT	506	92%	590	94.9%	1.00			
TT	44	8%	32	5.1%	**1.60(1.00-2.57)**	**0.049**	**1.61(1.00-2.58)**	**0.048**
C allele	793	72.1%	957	76.9%	1.00	-	-	-
T allele	307	27.9%	287	23.1%	**1.29(1.07-1.56)**	**0.007**	-	-

^a^The genotyping was successful in 550 cases and 622 controls for rs11225395 polymorphism;

Bold values are statistically significant (*P* < 0.05).

*Adjustment for sex and age.

### Stratified analysis of the rs11225395 polymorphism and CRC risk

Stratified analyses of gender, age, smoking, BMI, ESR, CRP, and drinking were conducted to measure the connection between the rs11225395 polymorphism and the risk of CRC (Table 3). We observed an increased risk for CRC patients among smokers, males, and those individuals aged ≥ 60 years. However, no positive findings were reported in the stratified analysis of drinking, BMI, ESR, and CRP.

**Table 3:** Stratified analyses between MMP-8 rs11225395 polymorphism and the risk of colorectal cancer.

**Variable**	**(case/control)**			**CT vs. CC**	**TT vs.CC**	**TT vs. CT+CC**	**TT+CT vs.CC**
	**CC**	**CT**	**TT**				
Sex							
Male	173/212	143/138	25/14	1.27(0.93-1.73); 0.129	**2.19(1.10-4.34); 0.025**	**1.98(1.01-3.87); 0.047**	**1.35(1.01-1.82); 0.046**
Female	114/155	76/85	19/18	1.22(0.82-1.80); 0.330	1.44(0.72-2.86); 0.304	1.33(0.68-2.61); 0.402	1.25(0.87-1.81); 0.229
Smoking							
Yes	161/194	130/123	23/12	1.14(0.83-1.58); 0.426	**2.31(1.12-4.79); 0.024**	**2.09(1.02-4.27); 0.040**	**1.37(1.00-1.87); 0.050**
No	126/173	89/100	21/20	1.11(0.77-1.60); 0.569	1.44(0.75-2.77); 0.273	1.38(0.73-2.62); 0.318	1.26(0.89-1.78); 0.192
Alcohol							
Yes	163/199	115/108	25/18	1.30(0.93-1.81); 0.124	1.70(0.89-3.22); 0.106	1.53(0.82-2.87); 0.181	1.36(0.99-1.86); 0.060
No	124/168	104/115	19/14	1.23(0.86-1.74); 0.259	1.84(0.89-3.81); 0.101	1.69(0.83-3.43); 0.151	1.29(0.92-1.81); 0.139
Age (years)							
<60	122/161	105/113	18/20	1.22(0.85-1.74); 0.275	1.18(0.60-2.33); 0.632	1.08(0.56-2.10); 0.814	1.23(0.86-1.70); 0.266
≥60	165/206	114/110	26/12	1.30(0.93-1.81); 0.122	**2.72(1.33-5.55); 0.006**	**2.46(1.22-4.97); 0.012**	**1.44(1.05-1.98); 0.025**
BMI							
<25	231/271	181/176	33/24	1.21(0.92-1.58);0.176	1.61(0.93-2.81);0.089	1.49(0.87-2.57);0.146	1.26(0.97-1.63);0.087
≥25	56/96	38/47	11/8	1.39(0.81-2.38);0.235	2.36(0.90-6.21);0.076	2.09(0.81-5.39);0.120	1.53(0.92-2.54);0.101
CRP, mg/dL							
<0.4	168/350	131/215	15/27	1.27(0.96-1.69);0.100	1.16(0.60-2.23);0.663	1.05(0.55-2.00);0.883	1.26(0.95-1.66);0.104
≥0.4	119/17	88/8	29/5	1.57(0.65-3.81);0.313	0.83(0.28-2.43);0.732	0.70(0.25-1.97);0.499	1.29(0.60-2.77);0.519
ESR, mm/hr							
<15	160/350	125/219	17/28	1.25(0.94-1.67);0.131	1.33(0.71-2.50);0.377	1.21(0.65-2.25);0.542	1.26(0.95-1.66);0.107
≥15	127/17	94/5	27/4	2.54(0.91-7.14);0.068	0.90(0.28-2.90);1.000	0.67(0.22-2.10);0.491	1.80(0.77-4.19);0.168

BMI: body mass index; CRP: C-reactive protein; ESR: erythrocyte sedimentation rate; Bold values are statistically significant (*P* < 0.05).

### Combined and interactive effects of the rs11225395 polymorphism and drinking or smoking on CRC risk

Due to the association of the rs11225395 polymorphism with environmental factors observed in Table 3, we next used cross-over analysis to further evaluate the impact of the interactions between the MMP-8 gene rs11225395 polymorphism and environmental factors on CRC susceptibility. Smokers with CT or TT genotype increased the risk of CRC when compared with non-smokers with CC genotype (Table 4). Similarly, CRC patients who carried the CT genotype and drank alcohol presented a higher risk of developing CRC than individuals who did not carry the CC genotype and drink alcohol (drinking + CT vs. non-drinking +CC: OR, 1.44, 95% CI, 1.02-2.05; *P* = 0.040). In summary, the data showed potential associations between the rs11225395 polymorphism and contributions from smoking or drinking to an increased risk of developing CRC.

**Table 4:** Genetic (G) and environmental (E) factors 2*4 fork analysis.

**G^a^**	**E^b^**	**Case**	**Control**	**OR (95%CI); P value**	**Reflecting information**
**rs11225395**					
TT vs. CC	Smoking				
+	+	23	12	**2.63(1.26,5.49); 0.008**	G, E combined effect
+	-	21	20	1.44(0.75,2.77); 0.271	G alone effect
-	+	161	194	1.14(0.84,1.55); 0.410	E alone effect
-	-	126	173	1.00 (reference)	Common control
CT vs. CC	Smoking				
+	+	130	123	**1.45(1.04,2.03); 0.030**	G, E combined effect
+	-	89	100	1.22(0.85,1.76); 0.283	G alone effect
-	+	161	194	1.14(0.84,1.55); 0.410	E alone effect
-	-	126	173	1.00 (reference)	Common control
TT vs. CC	Drinking				
+	+	25	18	1.88(0.98,3.60); 0.053	G, E combined effect
+	-	19	14	1.84(0.89,3.81); 0.097	G alone effect
-	+	163	199	1.11(0.81,1.52); 0.512	E alone effect
-	-	124	168	1.00 (reference)	Common control
CT vs. CC	Drinking				
+	+	115	108	**1.44(1.02,2.05); 0.040**	G, E combined effect
+	-	104	115	1.23(0.86,1.74); 0.258	G alone effect
-	+	163	199	1.11(0.81,1.52); 0.512	E alone effect
-	-	124	168	1.00 (reference)	Common control

^a^G (+): MMP8 gene rs11225395 variants (Heterozygous or homozygous); G (-): wild type; ^b^E(+): smoking/non-smoking; E(-): non-smoking/non-drinking; Bold values are statistically significant (*P* < 0.05).

### MMP-8 gene rs11225395 polymorphism and clinicopathological characteristics of CRC patients

Next, we assessed whether the MMP-8 gene rs11225395 polymorphism was connected to CRC clinicopathologic features (Table 5). The CRC patients with TT genotype were linked with TNM III+IV stage and lymph node metastasis. No positive association was observed for histological grade, tumor size, family history of CRC, histology, and location of CRC.

**Table 5:** The associations between MMP-8 rs11225395 polymorphism and clinical characteristics of colorectal cancer.

**Characteristics**	**Genotype distributions**			
	**CC**	**CT**	**TT**	**CT+TT**
Histological grade				
MD/WD	205/42	170/31	30/4	200/35
OR (95%CI); *P*-value	1.0 (reference)	1.12(0.68-1.87); 0.652	1.54(0.51-4.60); 0.439	1.17(0.72-1.91); 0.527
Histological grade				
PD/WD	40/42	18/31	10/4	28/35
OR (95%CI); *P*-value	1.0 (reference)	0.61(0.30-1.26); 0.179	2.63(0.76-9.05); 0.117	0.84(0.44-1.62); 0.604
Family History				
Yes/No	59/228	50/169	7/37	57/206
OR (95%CI); *P*-value	1.0 (reference)	1.14(0.75-1.75);0.538	0.73(0.31-1.72);0.472	1.07(0.71-1.61);0.749
TNM stage				
III+IV/I+II	164/123	118/101	34/10	152/111
OR (95%CI); *P*-value	1.0 (reference)	0.85(0.60-1.21); 0.369	**2.55(1.21-5.36); 0.011**	1.03(0.73-1.44); 0.877
Tumor size				
>5 cm/ ≤5 cm	174/113	120/99	26/18	146/117
OR (95%CI); *P*-value	1.0 (reference)	0.79(0.55-1.12); 0.188	0.94(0.49-1.79); 0.846	0.81(0.57-1.14); 0.225
Lymph node metastasis				
Yes/No	107/180	100/119	26/18	126/137
OR (95%CI); *P*-value	1.0 (reference)	1.41(0.99-2.02); 0.057	**2.43(1.27-4.64); 0.006**	**1.55(1.10-2.17); 0.012**
Histology				
Adenocarcinoma/others	269/18	212/7	42/2	254/9
OR (95%CI); *P*-value	1.0 (reference)	2.03(0.83-4.94); 0.114	1.41(0.32-6.28); 0.655	1.89(0.83-4.28); 0.122
Location of colorectal cancer				
colon cancer/ rectal cancer	99/188	73/146	9/35	82/181
OR (95%CI); *P*-value	1.0 (reference)	0.95(0.66-1.38); 0.785	0.49(0.23-1.06); 0.064	0.86(0.60-1.23); 0.408

Bold values are statistically significant (*P* <0.05). PD = Poorly differentiation, MD= Moderately differentiation, WD= Well differentiation.

### Genotype-based MMP-8 gene expression analysis and potential gene-gene interactions

**Figure 2:**
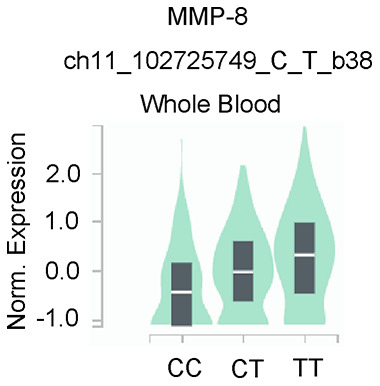
**Genotype-based mRNA expression alteration in whole blood for MMP-8 rs11225395 polymorphism based on data from the GTEx portal database (https://www.gtexportal.org/home).**

**Figure 3:**
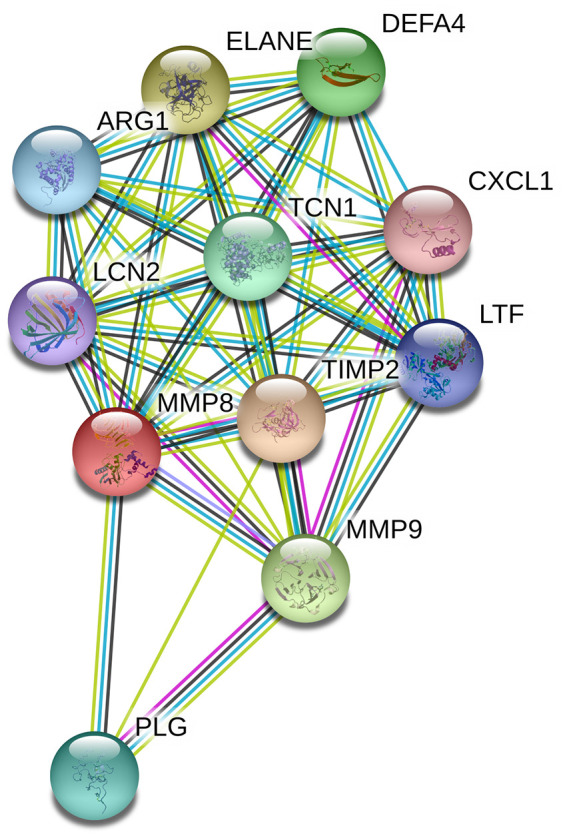
**Human MMP-8 interactions with other genes obtained from the String server.** The following genes participate in gene-gene interactions: TIMP2, Metalloproteinase inhibitor 2; ELANE, Neutrophil elastase; MMP9, Matrix metalloproteinase-9; DEFA4, Neutrophil defensin 4; TCN1, Transcobalamin-1; PLG, Plasminogen; ARG1, Arginase-1; LTF, Lactotransferrin; LCN2, Neutrophil gelatinase-associated lipocalin; CXCL1, Growth-regulated alpha protein.

Data from the GTEx portal uncovered that the rs11225395 polymorphism was shown to alter the expression of MMP-8 gene in whole-blood samples (*P* = 3.5e-17) (Figure 2). In addition, several genes including TIMP2, ELANE, MMP-9, DEFA4, TCN1, PLG, ARG1, LTF, LCN2, CXCL1 were involved in the interaction of MMP-8 (Figure 3), which was discovered by using the String online tool (http://string-db.org/).

### Survival analysis of CRC patients with the MMP-8 gene rs11225395 polymorphism

**Figure 4:**
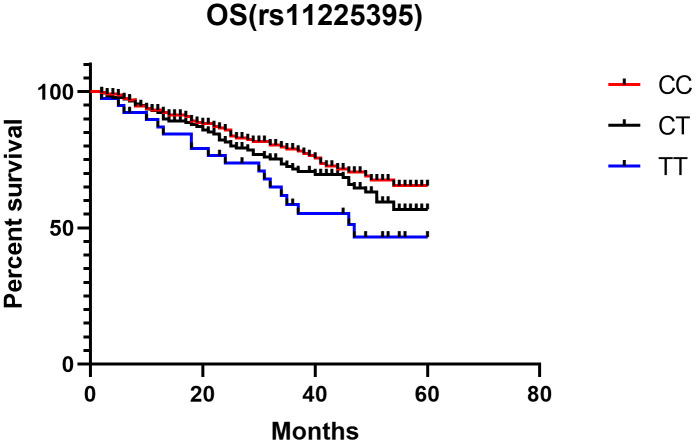
**Kaplan-Meier analysis of the association between MMP-8 rs11225395 polymorphism and overall survival of colorectal cancer patients.**

Last, Kaplan-Meier single factor analysis showed that CRC patients with TT genotype exhibited worse overall survival than those with a CC genotype (HR, 2.30, 95%CI, 1.19-4.43; log-rank *P* = 0.013, Figure 4).

## DISCUSSION

This study showed that the MMP-8 gene rs11225395 polymorphism was related with an elevated risk of CRC in a subpopulation of Chinese patients, especially among smokers, males, and those individuals aged ≥ 60 years. In addition, this polymorphism correlated with the occurrence of lymph node metastasis and TNM III+IV stage in CRC patients. Furthermore, TT genotype carriers showed worse overall survival with CC genotype carriers.

Recently, studies investigating the connection between the risk of cancers and rs11225395 polymorphism are increasingly emerging. Kubben et al. showed that the rs11225395 polymorphism was not related with gastric cancer risk and survival [23]. A subsequent study replicated the negative results obtained by Kubben et al., which revealed that the MMP-8 gene rs11225395 polymorphism was not related to hepatocellular carcinoma risk in a subpopulation of Han Chinese including 434 cases and 480 controls [26]. Nevertheless, an India study indicated that this SNP decreased the risk of bladder cancer in a population of 200 cases and 200 age-matched controls [28]. Unexpectedly, Debniak et al. from Poland uncovered that the rs11225395 polymorphism elevated the risk of malignant melanoma, while no association was detected between this SNP and breast cancer risk [20]. As for breast cancer, Hsiao et al. from Taiwan revealed that this SNP was not linked with breast cancer risk [21], but Wang et al. showed that rs11225395 polymorphism elevated the risk of breast cancer in a subpopulation of Han Chinese [30]. Other Taiwanese studies demonstrated no associations between the MMP-8 gene rs11225395 polymorphism and childhood leukemia [25], lung cancer [27], oral cancer [22], and bladder cancer risk [29]. Hashim et al. suggested that MMP-8 gene rs11225395 polymorphism was a protective factor associated with nasopharyngeal carcinoma susceptibility in a Malaysian population [24], and Arechavaleta-Velasco et al. detected that this locus increased the risk of ovarian cancer in Mexican women [31]. All such studies yielded conflicting findings regarding the association with this variant and cancer risk. There may be many reasons that may explain such contradictory findings: One, diverse exposure factors and dietary habits; two, different populations have genetic heterogeneity; three, the sample size among the aforementioned studies differed; four, the studies exhibited clinical heterogeneity among different cancers. To address these inconsistent results, a meta-analysis by Feng et al. to explore the connection between this SNP and cancer susceptibility, and found no association of the rs11225395 polymorphism and overall cancer risk [36]. However, elevated cancer risk was observed in non-Asian populations, and no relationship was detected in Asian populations [36]. Up to date, no studies investigated the link between CRC risk and the MMP-8 rs11225395 polymorphism. Therefore, we conducted this study to explore such association. Our study revealed that the MMP-8 rs11225395 polymorphism elevated the risk of CRC.

In addition, we uncovered an increased risk for developing CRC in males, smokers, and those subjects aged ≥ 60 years. However, Qiu et al. did not find any associations between hepatocellular carcinoma susceptibility and rs11225395 polymorphism when the analyses were stratified by age, gender, and drinking and smoking statuses [26]. Cross-over analysis showed that the integrated effects of the MMP-8 rs11225395 polymorphism and smoking or drinking were related to an increased risk of CRC. Our data also showed that the MMP-8 rs11225395 polymorphism related to TNM III+IV stage and lymph node metastasis among CRC patients.

Last, we explored the association between CRC prognosis and the MMP-8 rs11225395 polymorphism. We found that patients with TT genotype showed worse overall survival compared with patients with CC genotype. However, Velasco et al. indicated that the TT genetic carriers showed a poorer overall survival when compared with the CC + CT genotype carriers among ovarian cancer patients [31]. In addition, Kubben et al. observed that the MMP-8 gene rs11225395 polymorphism was not related to gastric cancer survival [23]. Decock et al. found that the rs11225395 polymorphism predicted a better overall survival in patients with breast cancer [19]. Different cancer tissues, disease stage, and clinical heterogeneity may contribute to these inconsistent findings. Further meta-analyses studying this issue are needed to clarify these contradictory results.

This study had some limitations. First, the sample size was relatively small, which may decrease the reliability of our results. Second, this case-control study failed to demonstrate a cause-effect relationship. Third, the current analysis only considered one polymorphism of the MMP8 gene. Fourth, other potential gene-gene interactions should be studied in future studies. Fifth, although the String online tool revealed an interaction between the MMP-8 gene and several other genes, we could not validate the endogenous expression of each candidate genes and whether their expression levels were linked to the survival of CRC patients. Sixth, we could not directly detect the MMP8 rs11225395 polymorphism site in clinical specimens. Last, why MMP-8 gene rs11225395 polymorphism was associated with the prognosis of CRC should be investigated. Nonetheless, our study here shows that the MMP-8 gene rs11225395 polymorphism correlates with the CRC risk and prognosis. Further studies in Chinese and other populations are warranted.

## METHODS

### Subjects

We enrolled 551 CRC cases and 623 controls from the First Hospital of Jilin University in this study. The diagnosis of CRC depended on pathological manifestations. The patients with digestive diseases, or CRC patients receiving chemotherapy or radiotherapy were excluded. Healthy controls were chosen from the same hospital. Written informed consent was got from each participant. Clinicopathological characteristics of all CRC patients were obtained from their medical records. All controls finished a standardized questionnaire including detailed clinical characteristics such age, sex, smoking and drinking. The Ethics Committees of the First Hospital of Jilin University approved this study; and it was in line with the standards of the Declaration of Helsinki.

### Genotyping

We collected 2 ml of peripheral blood from all CRC patients and matched controls, which were stored at -80° C until used. We used the TIANamp Blood DNA kit to obtain DNA (Tiangen Biotech, Beijing, China). Genotyping of the studied SNP was analyzed by RFLP-PCR. The primers used for this polymorphism were as following: GCCAGAGACTCAAGTGGGAGACTACCATGCAGATC (forward) and reverse primer TTATGATTGCCCAGACATTTG (reverse). Approximately 10% of all enrolled individuals were re-genotyped, and the concordance was 100% [32, 33].

### Genotype and gene expression correlation analysis

The data from the GTEx Portal database (https://www.gtexportal.org/home/) were utilized to evaluate the link between genotypes of the MMP-8 rs11225395 polymorphism and mRNA expression-level alteration [34].

### Statistical analysis

We used student’s *t*-test and chi-square (χ^2^) test to evaluate continuous and categorical variables, respectively. The Hardy-Weinberg equilibrium (HWE) test among controls was calculated by a goodness-of-fit χ^2^ test. Logistic regression analysis was utilized to estimate the odds ratios (ORs) and their 95% confidence intervals (CIs). The Kaplan-Meier method was used to calculate overall survival [35]. *P* value < 0.05 indicated a statistical difference. All statistical analyses were addressed by SPSS 22.0 (SPSS Inc., Chicago, USA).

### Availability of data and materials

The data can be made available by the corresponding author upon reasonable request.
